# Wheat GT-1-like Transcription Factor Boosts Cutin Biosynthesis

**DOI:** 10.3390/biom16081141

**Published:** 2026-08-05

**Authors:** Yuxi Shan, Minawar Yusup, Haoyu Li, Pengfei Zhi, Xiaoyu Wang, Jiao Liu, Cheng Chang

**Affiliations:** College of Life Sciences, Qingdao University, Qingdao 266071, China

**Keywords:** *Triticum aestivum*, cuticular lipid, long-chain acyl-CoA synthetase 2, transcriptional regulation

## Abstract

Cutin matrices in the cuticle cover plant epidermis, facilitating plant adaptation to stressful environments. Although cutin biosynthesis is extensively explored in the model plant *Arabidopsis thaliana*, the molecular mechanism governing cutin biosynthesis in the agriculturally important crop bread wheat (*Triticum aestivum* L.) remains largely unknown. The aim of the study is the characterization of the function and transcriptional regulation of a wheat gene involved in cutin biosynthesis. Long-chain acyl-CoA synthetase TaLACS2 was identified as an essential component of the wheat cutin biosynthetic machinery. Silencing of the wheat *TaLACS2* gene by barley stripe mosaic virus-induced gene silencing assay resulted in remarkably reduced cutin accumulation and increased cuticle permeability. Furthermore, wheat GT-1-like transcription factor TaGT-3b was identified as a positive regulator of cutin biosynthesis. Silencing of the wheat *TaGT-3b* gene led to significantly decreased cutin accumulation and enhanced cuticle permeability. Importantly, we found that TaGT-3b could occupy the promoter regions of the *TaLACS2* gene and that it functions as a transcriptional activator to activate *TaLACS2* gene transcription. Collectively, these results elucidated that wheat GT-1-like transcription factor TaGT-3b boosts cutin biosynthesis, probably by activating *TaLACS2* gene transcription, contributing to genetically improving cutin-associated traits in bread wheat.

## 1. Introduction

Staple crop bread wheat (*Triticum aestivum* L.) is widely employed for human food and animal feed [1]. Human population growth and excess consumption drive the demand for bread wheat [2]. However, environmental stresses severely affect wheat plant growth and seriously threaten wheat production [1]. As a hydrophobic skin, lipophilic cuticle covers wheat aerial organs like leaf blades, leaf sheaths, stems, and inflorescence [1,2]. Increasing evidence revealed that the cuticle contributes to plant resistance against abiotic and biotic stresses [3,4,5,6,7,8]. Therefore, exploring and exploiting cuticle biosynthesis facilitates wheat breeding for improved resistance against environmental stresses [6].

The hydrophobic property of the wheat cuticle is mainly conferred by cutin matrices and cuticular wax mixtures [9]. Wheat cutin matrices are insoluble in organic solvents and mainly comprise crosslinked polyesters of oxygenated fatty acids with C16 and C18 chain lengths [9]. Wheat cuticular wax mixtures are extractable by organic solvents and predominantly consist of alcohols, aldehydes, alkanes, fatty acids, alkyl esters, and ketones with above C20 (very-long-chain, VLC) chain lengths [10]. The accumulation of wheat cuticular lipid responds to the environmental cues like UV-B radiation [11]. Cutin biosynthesis mainly occurs in the endoplasmic reticulum of plant epidermal cells and is extensively explored in the dicot model plant *Arabidopsis thaliana* [9,10]. Long-chain acyl-coenzyme A synthases (LACSs) catalyze the esterifcation of C16 and C18 fatty acids to form the C16 and C18 acyl-CoAs, important precursors for the cutin biosynthesis [11,12,13]. In the ER, C16 and C18 acyl-CoAs are oxidized and acyltransferated by CYP86A and CYP77A family cytochrome P450 enzymes, HOTHEAD protein, as well as glycerol-3-phosphate acyltransferase enzymes to form monoacylglycerol cutin monomers [14,15,16,17]. These cutin monomers are then transported by trafficking pathways and transporter proteins to the cuticular region, where cutin monomers are polymerized into cross-linked cutin matrices [18,19,20,21,22,23,24,25,26].

In the dicot model plant Arabidopsis, a plethora of transcription factors are reported to regulate cutin biosynthesis [27,28,29]. For instance, TCP14 and TCP15 transcription factors positively regulate cutin biosynthesis by potentiating expression of the cutin biosynthesis genes *AtCYP86A4*, *AtGPAT6*, and *AtCUS2* [27]. Myeloblastosis (MYB) transcription factor AtMYB49 upregulates various cutin biosynthesis genes and positively regulates cutin deposition [28]. The MYB transcription factor AtMYB41 negatively regulates the cutin biosynthesis in Arabidopsis leaves [29]. However, cutin biosynthetic machinery and its regulatory mechanism are poorly understood in the agriculturally important crop bread wheat.

In this study, we aimed to characterize the function and transcriptional regulation of the wheat *long-chain acyl-CoA synthetase 2* (*TaLACS2*) gene in cutin biosynthesis. It was hypothesized that wheat long-chain acyl-CoA synthetase TaLACS2, resembling its homolog in Arabidopsis, plays a key role in cutin biosynthesis. To examine this hypothesis, we isolated the wheat long-chain acyl-CoA synthetase gene *TaLACS2*, characterized its roles in wheat cutin biosynthesis, and identified wheat GT-1-Like transcription factor TaGT-3b as the transcriptional regulator of *TaLACS2* gene.

## 2. Materials and Methods

### 2.1. Plant Materials and Growth Conditions

Wheat cultivar Yannong 999 was cultivated for the gene silencing, gene expression analysis, cutin quantification, leaf cuticle permeability measurement, and analysis of protein occupancy at gene promoters. Wheat seedlings that were 7 days old were kept in a growth chamber under a 16 h light photoperiod, a 21 °C day/18 °C night cycle, and 70% relative humidity. The water-soluble compound fertilizer Huawuque was employed for the fertilization of wheat plants.

### 2.2. Gene Expression Analysis

*TaGT-3b* and *TaLACS2* expression levels were measured by reverse transcription-quantitative polymerase chain reaction (RT-qPCR) assay, as described previously [30]. *TaGT-3b* and *TaLACS2* genes were analyzed using primers 5′AGTGGGGCGTGCAGGAGA3′/5′ATGTAATTCTTCAAAGAAG3′ and 5′ACACCGTGAAGGTGGGCGA3′/5′ACACTTGTTCATACGTTTG3′, under the PCR program: 95 °C for 2 min, 40 cycles at 95 °C for 20 s, 56 °C for 20 s, and 72 °C for 15 s, followed by 72 °C for 1 min. The housekeeping gene TaGADPH was analyzed using primers 5′ TTAGACTTGCGAAGCCAGCA3′/5′AAATGCCCTTGAGGTTTCCC3′ as a reference for RT-qPCR analysis.

### 2.3. Gene Silencing in Wheat Leaves

*TaGT-3b* and *TaLACS2* genes were silenced by barley stripe mosaic virus-induced gene silencing (BSMV-VIGS) assays, as described previously [31]. *TaGT-3b* and *TaLACS2* gene fragments were amplified using primers 5′AAGGAAGTTTAATATAGATCTTCTTGTAAGA3′/5′AACCACCACCACCGTGCAACAGCATGTCGATATG3′ and 5′AAGGAAGTTTAACATTGTGCAGTTCCCATC3′/5′AACCACCACCACCGTGTACATCCAGGATGAGCTG3′, respectively. About 2 weeks after virus inoculation, leaves of about 4-week-old plants with virus phenotypes were collected for the gene expression analysis, cutin quantification, and leaf cuticle permeability measurement.

### 2.4. Cutin Quantification and Leaf Cuticle Permeability Analysis

For the cutin accumulation and composition analysis, methyl esters of cutin monomers were extracted from delipidated and depolymerized leaf samples of 4-week-old BSMV-VIGS plants with dichloromethane as previously described [32,33,34]. Cutin constituents were quantified based on FID peak areas relative to internal standard per leaf area. A water loss and chlorophyll leaching assay analyzing cuticle permeability was conducted as previously described [32,33,34].

### 2.5. Measurement of Protein Occupancy on Gene Promoter Regions

The TaGT-3b coding region was amplified using the primers 5′GGGGACAAGTTTGTACAAAAAAGCAGGCTTCATGATGGAGGCGGGCGGAG3′/5′GGGGACCACTTTGTACAAGAAAGCTGGGTCTAGATCTTCTTGTAAGAGT3′, and cloned into the vector pCAMBIA1300-GFP to create the pCAMBIA1300-TaGT-3b-GFP. The *TaGT-3b* gene fragment was amplified using primers 5′GGGGACAAGTTTGTACAAAAAAGCAGGCTTCATGTATAGATCTTCTTGTAAGA3′/5′GGGGACCACTTTGTACAAGAAAGCTGGGTCGCAACAGCATGTCGATATG3′, and cloned into the vector pIPKb007 to create the RNAi-*TaGT-3b* construct. Wheat protoplast transfection and ChIP-qPCR were performed as previously described [32,33,34]. Promoter fragments of *TaLACS2-5A*, *TaLACS2-5B*, and *TaLACS2-5D* genes were analyzed by ChIP-qPCR using primers 5′TCAATACCAACACAAAAAC3′/5′GCTCATTATGTTGTCATGA3′, 5′GCAACAGAAACATGTGATG3′/5′TCTCGCATGTTTTAACTAC3′, 5′GGAGATGGCATCACGTTG3′/5′TGTAACACTTGAATTCGAG3′, 5′CATGCTATCAAAGAGCAAG3′/5′TTCATACACTGAGACGTTG3′, 5′GTGATATGCATTACCGAG3′/5′CCGAATACCGGAGGAACAC3′, 5′CTTAATATATGGCCTTTAG3′/5′TTTGGTATTAGATACATC3′, respectively.

### 2.6. Dual-Luciferase Reporter Assay

The Dual-Luciferase reporter assay analyzed the activation of *TaLACS2* promoters by TaGT-3b, as previously described [32,33,34]. The *TaLACS2-5A*, *TaLACS2-5B*, and *TaLACS2-5D* gene promoter regions were amplified using the primers 5′GGGGACAAGTTTGTACAAAAAAGCAGGCTTCGGCCACCACATCCTTCGTC3′/5′GGGGACCACTTTGTACAAGAAAGCTGGGTCCGAACCTTCCTCTCCAGCT3′, 5′GGGGACAAGTTTGTACAAAAAAGCAGGCTTCTCAAACTCCAAGCAAAACC3′/5′GGGGACCACTTTGTACAAGAAAGCTGGGTCCTTGGTGCCGTCAGATGCA3′, 5′GGGGACAAGTTTGTACAAAAAAGCAGGCTTCGATGCTCATATTGGTTCC3′/5′GGGGACCACTTTGTACAAGAAAGCTGGGTCCAGATGCAGATATGCTTTC3′, and cloned into the vectors 5XGAL4-LUC. Arabidopsis protoplast preparation and transfection, as well as a Dual-Luciferase reporter assay, were conducted as previously described [32,33,34].

### 2.7. Statistical Analysis

For the analysis of gene expression, cutin accumulation, leaf cuticle permeability, protein occupancy at gene promoters and Dual-Luciferase reporter assay data were analyzed using Student’s *t*-test, and values represent the mean ± standard deviation (** *p* < 0.01). These assays were repeated in three independent biological replicates using dependently prepared samples with similar results.

## 3. Results

### 3.1. Identification of Wheat Long-Chain Acyl-CoA Synthetase 2 (TaLACS2) Gene

**Figure 1:**
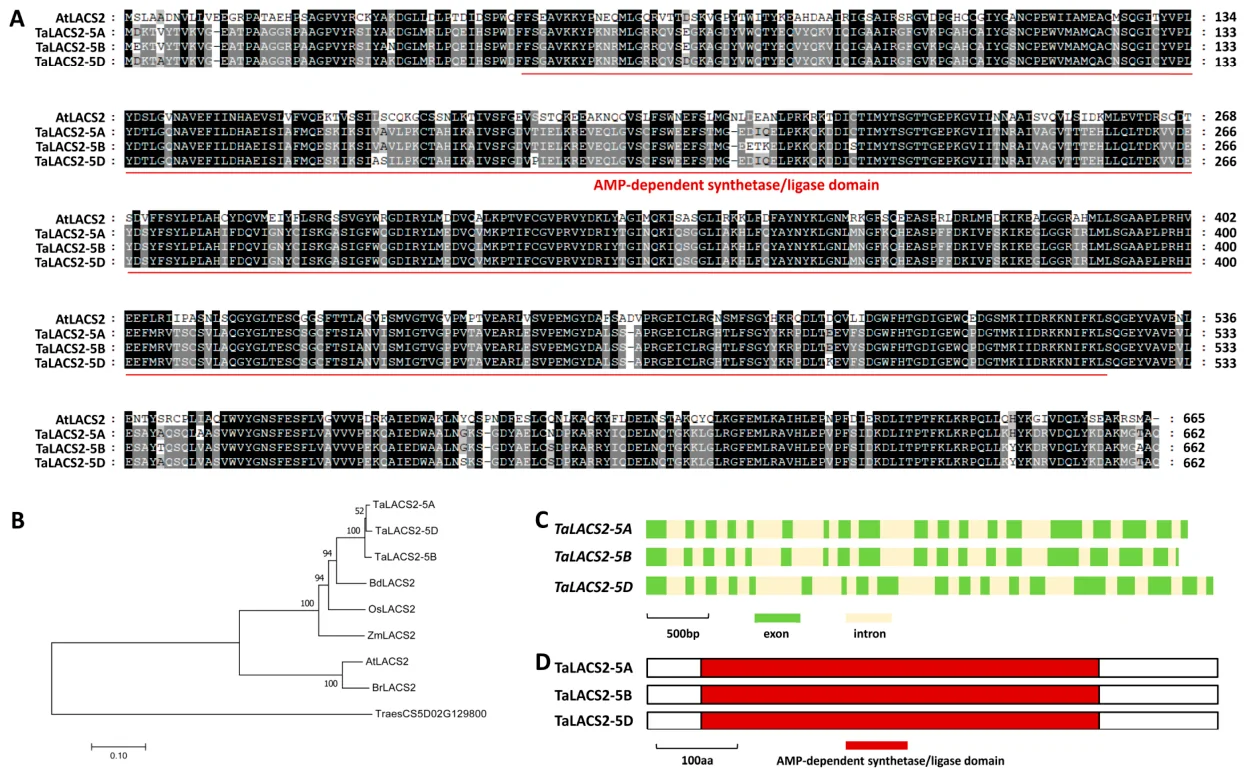
Sequence analysis of wheat TaLACS2. (**A**) Sequence alignment of wheat TaLACS2-5A, TaLACS2-5B, TaLACS2-5D, and Arabidopsis AtLACS2 proteins. AMP-dependent synthetase/ligase domain sequence was underlined with red. Identical residues among 4 protein sequences are shaded in dark, while residues conserved in at least 2 of the 4 proteins are shaded in gray. (**B**) Phylogenetic analysis of LACS2 proteins identified from bread wheat (*Triticum aestivum*, *Ta*), Japanese rice (*Oryza sativa* japonica, *Os*), maize (*Zea mays*, *Zm*), stiff brome (*Brachypodium distachyon*, *Bd*), *Arabidopsis thaliana* (*At*), and field mustard (*Brassica rapa*, *Br*). Neighbor-joining tree was examined with 1000 bootstrap replicates. (**C**) Organization of exons and introns in the genomic sequences of wheat *TaLACS2* genes. (**D**) Arrangement of domains in wheat TaLACS2 proteins.

Arabidopsis long-chain acyl-CoA synthetase 2 (AtLACS2) gets involved in cutin biosynthesis [11]. In this study, we employed the Arabidopsis AtLACS2 (At1g49430) sequence as a query to search against the wheat genome database and obtained highly homologous *TaLACS2-5A* (*TraesCS5A02G411500*), *TaLACS2-5B* (*TraesCS5B02G415100*), and *TaLACS2-5D* (*TraesCS5D02G420300*) from the wheat 5A, 5B, and 5D chromosomes. Wheat TaLACS2-5A, TaLACS2-5B, and TaLACS2-5D proteins share 62% amino acid sequence identity with Arabidopsis AtLACS2 protein (Figure 1A). Phylogenetic analysis confirmed that the TaLACS2-5A, TaLACS2-5B, and TaLACS2-5D proteins are close homologs of rice OsLACS2, maize ZmLACS2, stiff brome BdLACS2, *Arabidopsis* AtLACS2, and field mustard BrLACS2 (Figure 1B). The genome sequences of allelic *TaLACS2-5A*, *TaLACS2-5B*, and *TaLACS2-5D* genes contain nineteen exons and eighteen introns (Figure 1C). AMP-dependent synthetase/ligase domain exists in the TaLACS2-5A, TaLACS2-5B, and TaLACS2-5D proteins (Figure 1D).

### 3.2. Functional Analysis of TaLACS2 in Wheat Cutin Biosynthesis

**Figure 2:**
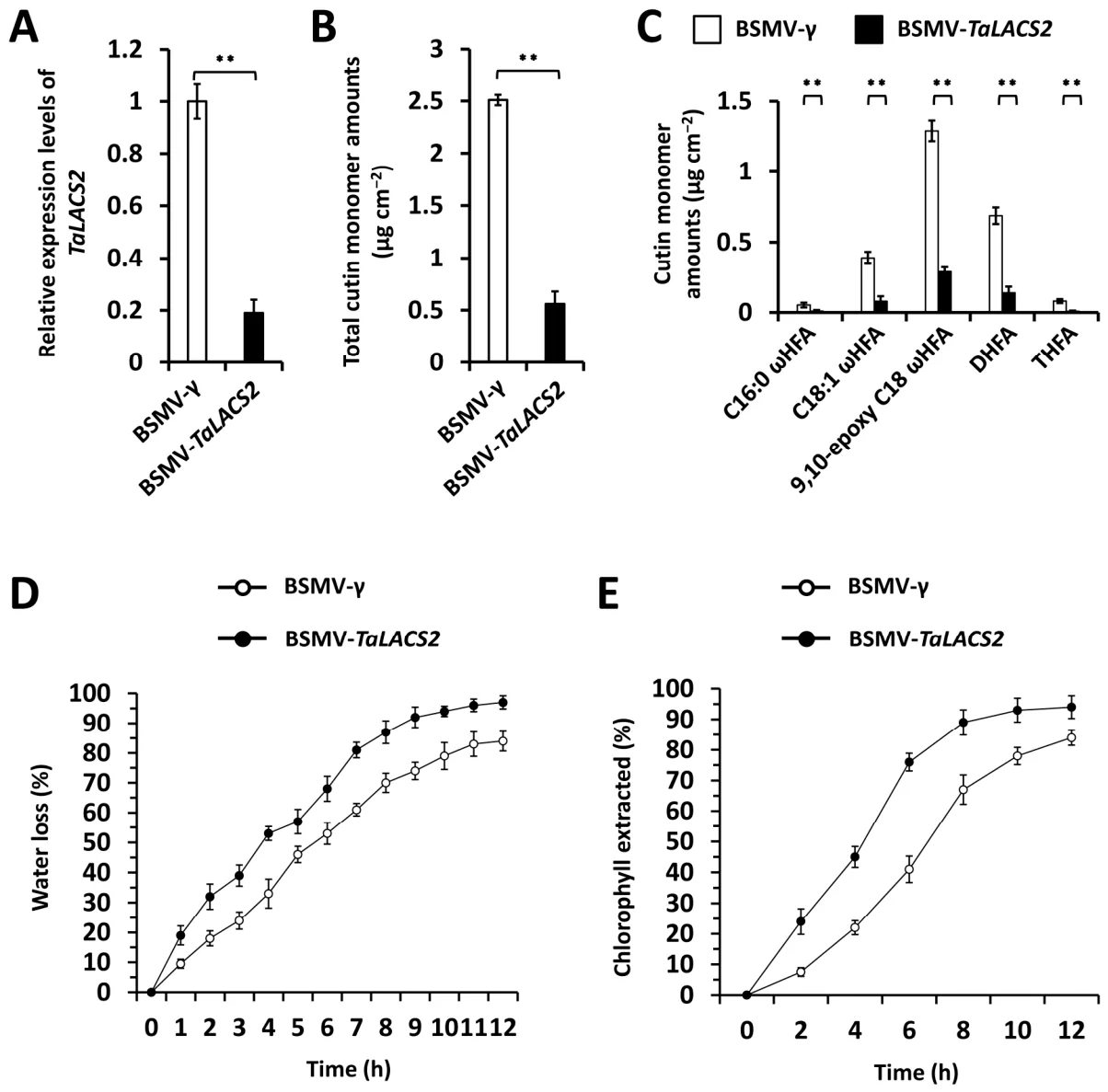
Functional analysis of the *TaLACS2* gene in wheat cutin biosynthesis. (**A**) Relative accumulation levels of *TaLACS2* gene transcripts in the wheat leaves where the *TaLACS2* gene was silenced. (**B**) Total cutin monomer amounts in the wheat leaves where the *TaLACS2* gene was silenceds. (**C**) Amounts of cutin monomers 16-hydroxy-hexadecanoic acid (C16:0 ωHFA), 18-hydroxy-octadec-9-enoic acid (C18:1 ωHFA), 9,10-epoxy 18-hydroxy-octadecanoic acid (9,10-epoxy C18 ωHFA), 9(10), 16-dihydroxy-hexadecanoic acid (DHFA) and 9,10,18-trihydroxy-octadecanoic acid (THFA) in the wheat leaves where the *TaLACS2* gene was silenced. (**D**) Rates of water loss and (**E**) chlorophyll leaching in wheat leaves where the *TaLACS2* gene was silenced. Data were statistically analyzed using Student’s *t*-test (** *p* < 0.01).

To examine the function of the *TaLACS2* gene in wheat cutin biosynthesis, we silenced the *TaLACS2* gene using a BSMV-VIGS assay in the wheat plants. An RT-qPCR assay validated that *TaLACS2* gene transcript accumulation levels significantly decreased in the wheat leaves where the *TaLACS2* gene was silenced (Figure 2A). We performed the GC-MS assay to measure the cutin accumulation in these BSMV-VIGS wheat leaves. As shown in Figure 2B, knockdown of *TaLACS2* gene expression resulted in a decrease in cutin accumulation to 22.3%. Accumulation levels of cutin monomers significantly decreased in the wheat leaves where the *TaLACS2* gene was silenced, compared with BSMV-γ control leaves (Figure 2C). These results indicated that the wheat long-chain acyl-CoA synthetase TaLACS2 gets involved in the wheat cutin biosynthesis. We then characterized the effect of the *TaLACS2* gene on wheat leaf cuticle permeability. As shown in Figure 2D,E, knockdown of *TaLACS2* gene expression resulted in enhanced water loss and chlorophyll leaching rates—compared with BSMV-γ control leaves—indicating that the *TaLACS2* gene is essential for wheat leaf cuticle impermeability. These data collectively support that wheat long-chain acyl-CoA synthetase TaLACS2 plays a key role in cutin biosynthesis and contributes to the cuticle barrier property.

### 3.3. Identification of Wheat GT-1-like Transcription Factor TaGT-3b

**Figure 3:**
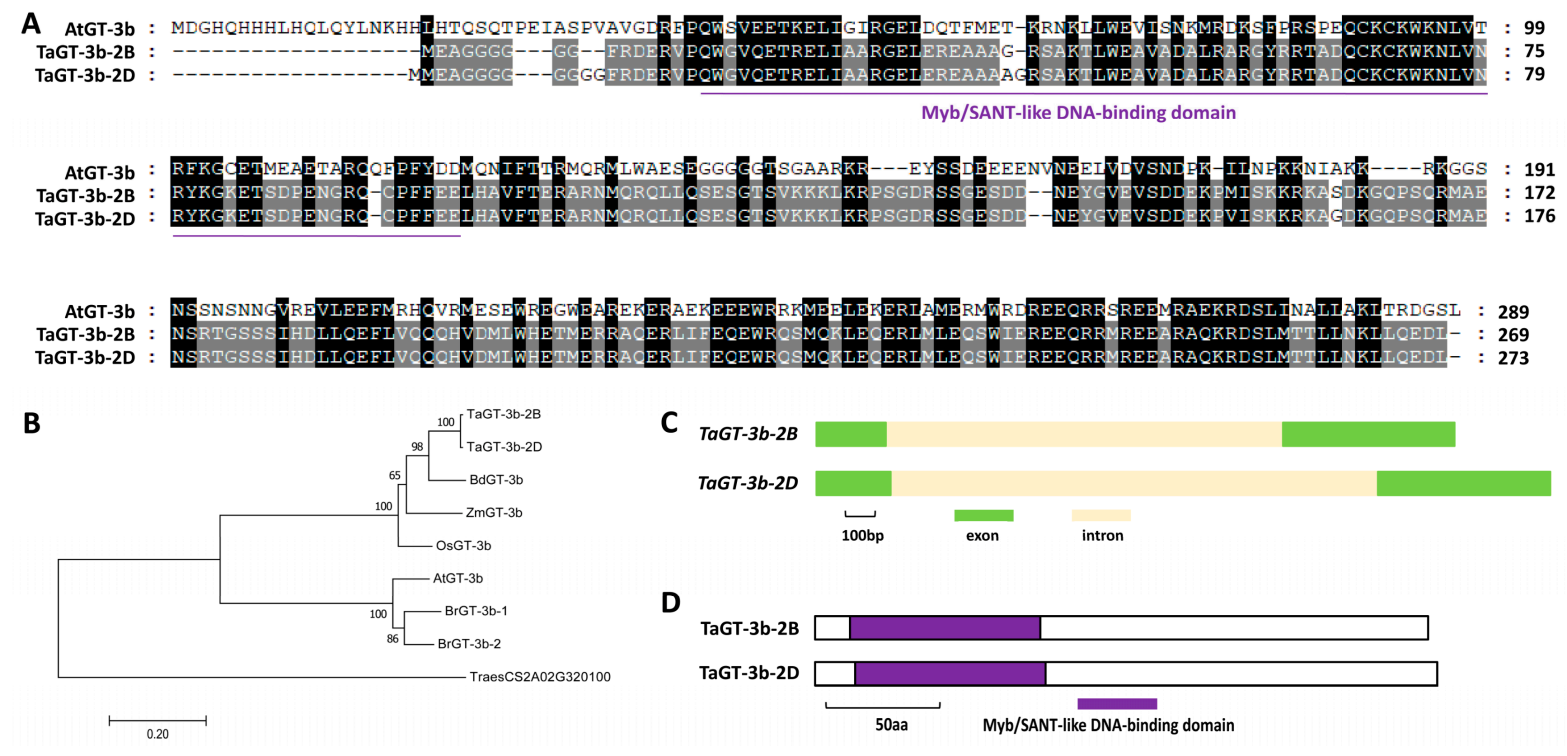
Sequence analysis of wheat TaGT-3b. (**A**) Sequence alignment of wheat TaGT-3b-2B, TaGT-3b-2D, and Arabidopsis AtGT-3b proteins. Myb/SANT-like DNA-binding domain was underlined with purple. Identical residues among 3 protein sequences are shaded in dark, while residues conserved in at least 2 of the 3 proteins are shaded in gray. (**B**) Phylogenetic analysis of GT-3b proteins identified from bread wheat (*Triticum aestivum*, *Ta*), Japanese rice (*Oryza sativa* japonica, *Os*), maize (*Zea mays*, *Zm*), stiff brome (*Brachypodium distachyon*, *Bd*), *Arabidopsis thaliana* (*At*), and field mustard (*Brassica rapa*, *Br*). Neighbor-joining tree was examined with 1000 bootstrap replicates. (**C**) Organization of exons and introns in the genomic sequences of wheat *TaGT-3b* genes. (**D**) Arrangement of domains in wheat TaGT-3b proteins.

Sequence analysis showed that the *GT-1* cis-element (*GAAAAA*) exists in the promoter regions of the *TaLACS2-5A*, *TaLACS2-5B*, and *TaLACS2-5D* genes. Previous studies revealed that Arabidopsis GT-1-like transcription factor AtGT-3b could directly recognize the *GT-1* cis-element (*GAAAAA*) in order to regulate calmodulin gene *SCaM-4* [35]. Herein, we employed the Arabidopsis AtGT-3b (At2g38250) sequence as a query to search against the wheat genome database and obtained highly homologous *TaGT-3b-2B* (*TraesCS2B02G457200*) and *TaGT-3b-2D* (*TraesCS2D02G433900*) from the wheat 2B and 2D chromosomes. The wheat TaGT-3b-2B and TaGT-3b-2D proteins share 37% amino acid sequence identity with the Arabidopsis AtGT-3b protein (Figure 3A). Phylogenetic analysis confirmed that the wheat TaGT-3b-2B and TaGT-3b-2D proteins are close homologs of rice OsGT-3b, maize ZmGT-3b, stiff brome BdGT-3b, *Arabidopsis* AtGT-3b, and field mustard BrGT-3b (Figure 3B). Genome sequences of allelic *TaGT-3b-2B* and *TaGT-3b-2D* genes contain two exons and one intron (Figure 3C). Myb/SANT-like DNA-binding domain exists in the N-terminal parts of TaGT-3b-2B and TaGT-3b-2D proteins (Figure 3D).

### 3.4. Functional Analysis of TaGT-3b in Wheat Cutin Biosynthesis

**Figure 4:**
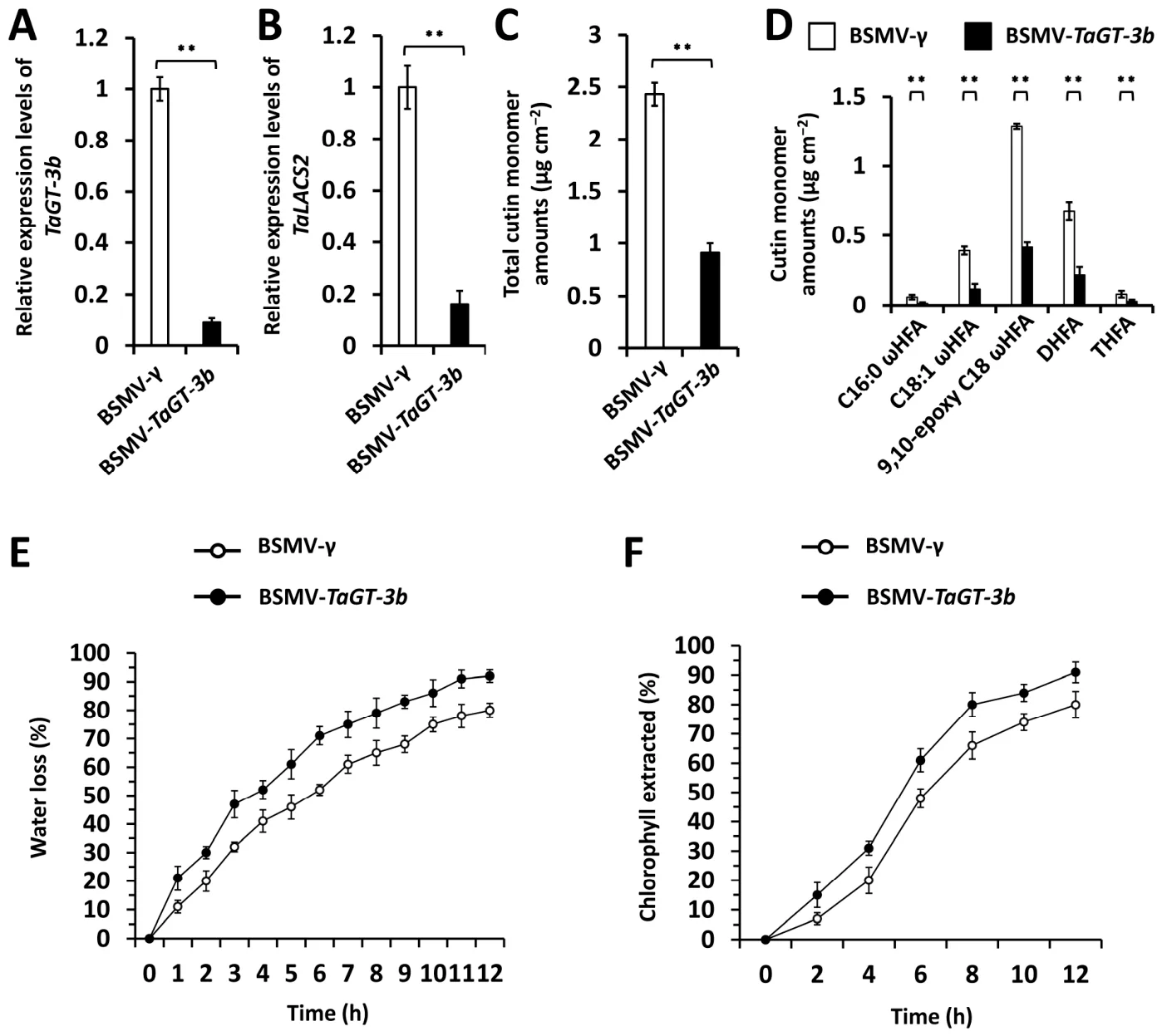
Functional analysis of *TaGT-3b* gene in wheat cutin biosynthesis. (**A**) Relative accumulation levels of *TaGT-3b* gene transcripts in the wheat leaves with silenced *TaGT-3b* genes. (**B**) RT-qPCR analysis of *TaLACS2* gene transcripts accumulation levels in the wheat leaves with silenced *TaGT-3b* gene. (**C**) Total cutin monomer amounts in the wheat leaves with silenced *TaGT-3b* genes. (**D**) Amounts of cutin monomers 16-hydroxy-hexadecanoic acid (C16:0 ωHFA), 18-hydroxy-octadec-9-enoic acid (C18:1 ωHFA), 9,10-epoxy 18-hydroxy-octadecanoic acid (9,10-epoxy C18 ωHFA), 9(10), 16-dihydroxy-hexadecanoic acid (DHFA) and 9,10,18-trihydroxy-octadecanoic acid (THFA) in the wheat leaves with silenced *TaGT-3b* genes. (**E**) Rates of water loss and (**F**) chlorophyll leaching in wheat leaves with silenced *TaGT-3b* genes. Data were statistically analyzed using Student’s *t*-test (** *p* < 0.01).

To examine the function of the *TaGT-3b* gene in wheat cutin biosynthesis, we silenced the *TaGT-3b* gene using BSMV-VIGS in wheat plants. An RT-qPCR assay validated that *TaGT-3b* gene transcript accumulation levels significantly decreased in the wheat leaves with a silenced *TaGT-3b* gene (Figure 4A). Notably, silencing of the *TaGT-3b* gene resulted in a significant reduction in the accumulation levels of the *TaLACS2* gene transcript (Figure 4B). We performed the GC-MS assay to measure the cutin accumulation in these BSMV-VIGS wheat leaves. As shown in Figure 4C, knockdown of *TaGT-3b* gene expression resulted in a decrease in cutin accumulation to 37.4%. Accumulation levels of cutin monomers C16:0 ωHFA, C18:1 ωHFA, 9,10-epoxy C18 ωHFA, DHFA, and THFA significantly decrease in the wheat leaves with silenced *TaGT-3b* genes compared with BSMV-γ control leaves (Figure 4D). These results indicate that the wheat GT-1-like transcription factor TaGT-3b positively regulates cutin biosynthesis. We then characterized the effect of the *TaGT-3b* gene on wheat leaf cuticle permeability. As shown in Figure 4E,F, knockdown of *TaGT-3b* gene expression resulted in enhanced water loss and chlorophyll leaching rates—compared with BSMV-γ control leaves—indicating that the *TaGT-3b* gene positively contributes to wheat leaf cuticle impermeability. These data collectively support that wheat GT-1-like transcription factor TaGT-3b positively regulates the cutin biosynthesis essential for the cuticle barrier property.

### 3.5. Regulation of Cutin Biosynthesis Gene TaLACS2 by GT-1-like Transcription Factor TaGT-3b

**Figure 5:**
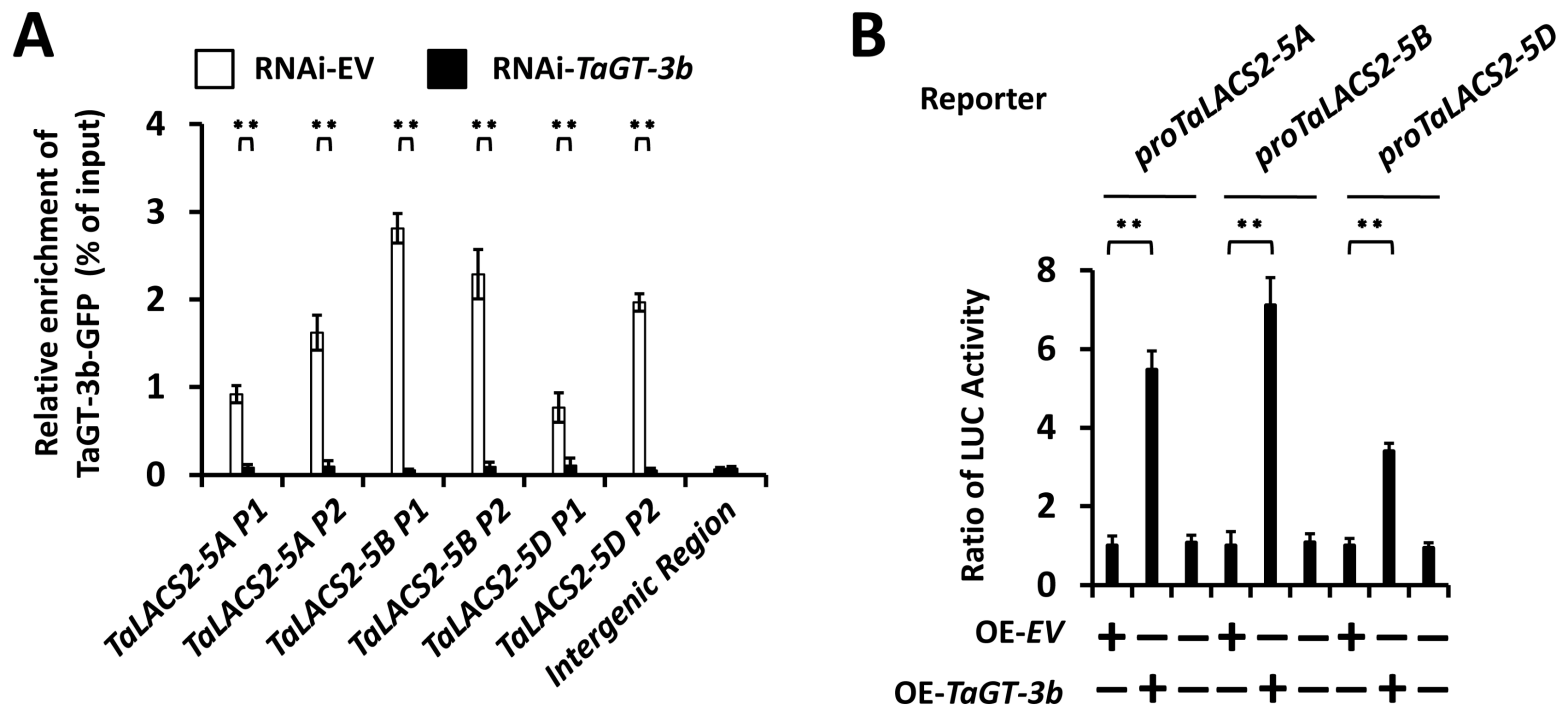
Characterization of TaGT-3b regulation on *TaLACS2* gene transcription. (**A**) ChIP-qPCR analysis of TaGT-3b occupancy on *TaLACS2* promoter regions. Gene promoter fragments chosen for the ChIP-qPCR analysis were represented with P1 and P2. EV in the RNAi-EV indicates empty vector. (**B**) Dual-Luciferase reporter analysis of TaGT-3b activation on the transcription driven by *TaLACS2* promoters. Data were statistically analyzed using Student’s *t*-test (** *p* < 0.01).

To analyze the potential direct regulation of wheat GT-1-like transcription factor TaGT-3b on *TaLACS2* gene transcription, we first transfected the wheat protoplast cells with the TaGT-3b-GFP and indicated RNAi constructs and conducted a ChIP-qPCR assay to examine the occupancy of TaGT-3b-GFP at the *TaLACS2* promoter regions. In the ChIP-qPCR assay, an intergenic region was employed as the negative control, and wheat protoplast cells co-expressing RNAi-*TaGT-3b* vector were included as another negative control. ChIP-qPCR analysis demonstrated that DNA fragments of *TaLACS2-5A*, *TaLACS2-5B*, and *TaLACS2-5D* gene promoters were found to be immuno-precipitated with the antibody against TaGT-3b-GFP, indicating that wheat GT-1-like transcription factor TaGT-3b enriches at the *TaLACS1* promoter regions (Figure 5A). We conducted the Dual-Luciferase reporter assay to analyze the regulation of GT-1-like transcription factor TaGT-3b on the gene transcription driven by *TaLACS2* promoters. As shown in Figure 5B, expression of the *TaGT-3b* gene resulted in the increase in relative LUC activity to above 3.4 compared with 1 for the empty vector control, indicating that wheat GT-1-like transcription factor TaGT-3b directly activates the gene transcription driven by *TaLACS2* gene promoters (Figure 5B). These findings collectively support that wheat GT-1-like transcription factor TaGT-3b activates cutin biosynthesis gene *TaLACS2* transcription.

## 4. Discussion

### 4.1. Long-Chain Acyl-CoA Synthetase TaLACS2 Is an Essential Component of Wheat Cutin Biosynthetic Machinery

Plant long-chain acyl-CoA synthetase widely gets involved in lipid biosynthesis, fatty acid catabolism and transport [11]. Arabidopsis *atlacs2* null mutant plants showed slightly increased cuticular wax accumulation but significantly decreased cutin deposition [11]. Leaves of Arabidopsis *atlacs2* null mutant plants exhibited faster chlorophyll release and displayed defects in the cuticular barrier [11]. In vitro assays supported that Arabidopsis AtLACS2 isozyme catalyzes the synthesis of ω-hydroxy fatty acyl-CoA intermediates in cutin biosynthesis [1,2,3,4,5]. Interestingly, the expression of *Glycine max GmLACS2-3* in an Arabidopsis *atlacs2* mutant greatly suppressed its phenotype, and overexpression of *GmLACS2-3* in wild-type plants significantly increased cutin amounts, indicating the conserved role of *Glycine max GmLACS2-3* in cutin synthesis [36]. Herein, we examined the function of wheat long-chain acyl-CoA synthetase TaLACS2 in cutin biosynthesis. Total cutin loads, as well as the amounts of major cutin monomers C16:0 ωHFA, C18:1 ωHFA, 9,10-epoxy C18 ωHFA, DHFA, and THFA, significantly decreased in the *TaLACS2*-silenced wheat leaves. Faster water loss and chlorophyll leaching were observed on the wheat leaves where the *TaLACS2* gene was silenced. These studies imply that the essential role of the *LACS2* gene in cutin biosynthesis is evolutionarily conserved among dicots and monocots.

### 4.2. Wheat GT-1-like Transcription Factor TaGT-3b Positively Regulates Cutin Biosynthesis

Arabidopsis GT-1-like transcription factor, AtGT-3b, was isolated as a transcriptional regulator of calmodulin gene *SCaM-4* and responsible for pathogen- and salt-induced *SCaM-4* gene expression in soybean and Arabidopsis [35]. AtGT-3b could directly interact with the GT-1 cis-element *GAAAAA*, a core cis-acting element for the induction of the *SCaM-4* gene [35]. Herein, we identified the wheat GT-1-like transcription factor TaGT-3b and characterized its roles in wheat cutin biosynthesis. Total cutin loads and the accumulation of major cutin monomers C16:0 ωHFA, C18:1 ωHFA, 9,10-epoxy C18 ωHFA, DHFA, and THFA significantly decrease in the *TaGT-3b*-silenced wheat leaves. Faster water loss and chlorophyll leaching were observed in the wheat leaves with silenced *TaGT-3b* genes. These results indicated that GT-1-like transcription factor TaGT-3b positively regulates cutin biosynthesis.

Arabidopsis GT-1-like transcription factor AtGT-3b could directly recognize the *GT-1* cis-element (*GAAAAA*) to regulate the calmodulin gene *SCaM-4* [35]. Herein, we showed that silencing of the *TaGT-3b* gene resulted in a significant reduction in *TaLACS2* gene transcript accumulation levels. A ChIP-qPCR assay demonstrated that wheat GT-1-like transcription factor TaGT-3b enriches at *TaLACS1* promoter regions. The Dual-Luciferase reporter assay revealed that wheat GT-1-like transcription factor TaGT-3b could stimulate the gene transcription driven by the *TaLACS2* promoters. These results collectively support that wheat GT-1-like transcription factor TaGT-3b activates transcription of the *long-chain acyl-CoA synthetase TaLACS2* gene to boost cutin biosynthesis. Notably, knockdown of *TaLACS2* gene expression resulted in a decrease in cutin accumulation to 22.3%, while knockdown of *TaGT-3b* gene expression resulted in a decrease in cutin accumulation to 37.4%, suggesting that more than one transcriptional activator stimulates the *TaLACS2* gene. Identifying other transcriptional regulators of the *TaLACS2* gene could expand our understanding of cutin biosynthesis.

Previous studies revealed that cuticular lipid biosynthesis is regulated by various transcription factors in bread wheat [30,31,32,33,34,35,36,37,38]. For instance, wheat MYB transcription factors TaEPBM1, TaMYB30, and TaMYB46 positively regulate cuticular wax biosynthesis by activating the transcription of wax biosynthesis genes like *TaECR*, *TaKCR1*, *TaKCS1*, *TaKCS2*, *TaCER3*, and *TaLACS1* [30,32,33,34]. Interestingly, the expression of transcription factor genes *TaEPBM1* and *TaMYB30* could respond to the environmental cues of air humidity and UV-B radiation, thereby mediating the humidity and UV-B-reponsive biosynthesis of cuticular wax [32,33,34]. Transcription of the Arabidopsis *AtGT-3b* gene is rapidly induced by treatment with the pathogen *Pseudomonas syringae* pv. *tomato* DC3000 or with 150 mM NaCl [35]. Therefore, it is intriguing to examine the potential roles of the wheat *TaGT-3b* gene in wheat resistance against pathogen infections and salt stress.

### 4.3. Genetic Manipulation of TaLACS2 and TaGT-3b Represent a New Avenue for Wheat Resistance Breeding

As discussed in previous reviews, exploiting cuticle biosynthesis could contribute to crop stress resistance improvement [1,2,3,4,5]. Allelic analysis of *TaLACS2* and *TaGT-3b* genes in wheat cultivars and the identification of *TaLACS2* and *TaGT-3b* elite haplotypes could contribute to the marker-assisted selection (MAS)-based wheat genetic improvement for stress resistance.

Increasing evidence indicates that cutin biosynthesis gets involved in plant developmental events [7]. In this study, we employed a BSMV-VIGS technique to transiently silence the *TaLACS2* and *TaGT-3b* genes to specifically characterize their function in wheat cutin biosynthesis. Generating stable wheat mutants of the *TaLACS2* and *TaGT-3b* genes using CRISPR-Cas9 or the TILLING technique might provide more insight into their functions in wheat development and facilitate their proper employment in wheat breeding [37,38,39,40,41].

## 5. Conclusions

Herein, we identified the GT-1-like transcription factor TaGT-3b as a positive regulator of wheat cutin biosynthesis and demonstrated that TaGT-3b activates transcription of the wheat cutin biosynthesis gene *TaLACS2* to boost cutin biosynthesis. Knockdown of *TaGT-3b* and *TaLACS2* gene expression resulted in attenuated cutin accumulation. TaGT-3b could occupy the promoter regions of the *TaLACS2* gene and functions as a transcriptional activator to activate *TaLACS2* gene transcription. These results collectively elucidated that GT-1-like transcription factor TaGT-3b boosts cutin biosynthesis, probably by activating *TaLACS2* gene transcription, facilitating the genetic improvement of wheat cutin-associated traits for climate-smart agriculture.

## Data Availability

The data presented here are available upon request from correspondence.
